# Repression of c-Kit by p53 is mediated by miR-34 and is associated with reduced chemoresistance, migration and stemness

**DOI:** 10.18632/oncotarget.1202

**Published:** 2013-08-06

**Authors:** Helge Siemens, Rene Jackstadt, Markus Kaller, Heiko Hermeking

**Affiliations:** ^1^ Experimental and Molecular Pathology, Institute of Pathology, Ludwig-Maximilians-University München, D-80337 Munich, Germany; ^2^ German Cancer Consortium (DKTK), D-69120 Heidelberg, Germany; ^3^ German Cancer Research Center (DKFZ), D-69120 Heidelberg, Germany

**Keywords:** p53, miR-34a, miR-34b/c, c-Kit, migration, chemoresistance, stemness

## Abstract

The c-Kit receptor tyrosine kinase is commonly over-expressed in different types of cancer. p53 activation is known to result in the down-regulation of c-Kit. However, the underlying mechanism has remained unknown. Here, we show that the p53-induced miR-34 microRNA family mediates repression of c-Kit by p53 via a conserved seed-matching sequence in the c-*Kit* 3'-UTR. Ectopic miR-34a resulted in a decrease in Erk signaling and transformation, which was dependent on the down-regulation of c-Kit expression. Furthermore, ectopic expression of c-Kit conferred resistance of colorectal cancer (CRC) cells to treatment with 5-fluorouracil (5-FU), whereas ectopic miR-34a sensitized the cells to 5-FU. After stimulation with c-Kit ligand/stem cell factor (SCF) Colo320 CRC cells displayed increased migration/invasion, whereas ectopic miR-34a inhibited SCF-induced migration/invasion. Activation of a conditional c-*Kit* allele induced several stemness markers in DLD-1 CRC cells. In primary CRC samples elevated c-Kit expression also showed a positive correlation with markers of stemness, such as *Lgr5*, *CD44*, *OLFM4*, *BMI-1* and *β-catenin*. On the contrary, activation of a conditional *miR-34a* allele in DLD-1 cells diminished the expression of c-Kit and several stemness markers (*CD44*, *Lgr5* and *BMI-1*) and suppressed sphere formation. MiR-34a also suppressed enhanced sphere-formation after exposure to SCF. Taken together, our data establish c-Kit as a new direct target of miR-34 and demonstrate that this regulation interferes with several c-Kit-mediated effects on cancer cells. Therefore, this regulation may be potentially relevant for future diagnostic and therapeutic approaches.

## INTRODUCTION

c-Kit, which is also known as CD117 or stem cell factor receptor, is a type III receptor tyrosine kinase (RTK) and an important mediator/initiator of several signaling cascades. The c-*Kit* gene was initially identified as the cellular homolog of v-*kit*, the oncogene of the Hardy-Zuckerman 4 feline sarcoma virus [1, 2]. Binding of its ligand stem cell factor (SCF, also known as steel factor) leads to receptor homodimerization and autophosphorylation of specific tyrosine residues on the intracellular domain of the receptor, which activates several signaling cascades [3]. Among these are the PI-3, Src, Jak/Stat, and MAP kinase induced pathways, as well as phospholipase C and D mediated signaling [4]. c-Kit exerts numerous functions in hematopoiesis, pigmentation, and gametogenesis and is involved in T-cell differentiation and mast cell regulation as well [4-8]. Deregulated expression and activation of c-Kit contributes to several types of diseases among them cancer [4]. For example, the c-Kit signaling axis is involved in leukemia [9, 10], glioblastoma [11], melanoma [12, 13], lung [14, 15] and breast cancer [16]. However, the role of c-Kit expression in colorectal cancer (CRC) is still largely unknown (see also discussion). Aberrant activation of c-Kit is associated with diminished chemo-responsiveness/chemoresistance of cancer cells and increased oncogenic signaling (e.g. in gastrointestinal stromal tumors [GISTs]) presumably by mediating an escape from apoptotic triggers [17-21]. The ligand of c-Kit, SCF/stem cell factor, is often expressed at elevated levels in tumor cells as well and contributes to the autocrine/paracrine transmission of oncogenic signals [22-25].

Interestingly, the microRNAs (miRNAs) miR-193a, −193b, −221, −222 and −494 are known to repress the expression of c-Kit by directly targeting its mRNA [26-29]. MiRNAs are small non-protein-coding RNAs of ~20-25 nucleotides length, which repress target mRNAs by binding to their 3'-untranslated regions (3'-UTRs) [30].

The *p53* tumor suppressor gene encodes a transcription factor, which is activated by numerous cellular stresses, which generally lead to DNA damage [31]. Interestingly, a p53-dependent down-regulation of c-Kit expression has been observed in mice, which occurred in the absence of direct binding of p53 to the c-*Kit* promoter [32]. Recently, microRNAs have been implicated in the repression of genes by p53 [33]. Among the most prominently p53-induced miRNAs, are the members of the miR-34 family: miR-34a, miR-34b and miR-34c, which are encoded by two different genes [34]. miR-34a/b/c were found to mediate several different tumor suppressive activities of p53, e.g. cell cycle arrest, as well as inhibition of stemness, induced pluripotent stem-cells (IPS), epithelial-mesenchymal transition (EMT)/metastasis and metabolism [33]. In addition, miR-34 genes may also be involved in other physiological processes, as for example in aging of the heart [35].

Here we report that miR-34 directly targets the c-*Kit* mRNA and thereby mediates repression of c-*Kit* expression by p53. Accordingly, miR-34 activation negatively regulated c-Kit mediated signaling events and cell transformation. Furthermore, miR-34a-mediated chemosensitization was accompanied by down-regulation of c-Kit. In addition, SCF-induced migration and invasion was abrogated by ectopic miR-34. Ectopic expression of c-Kit in CRC lines enhanced the expression of numerous markers of stemness, which was in agreement with an association of elevated c-Kit expression in primary CRC tumors and the expression of stemness markers, such as *OLFM4*, *LGR5*, *BMI-1* and *CD44*, whereas ectopic expression of miR-34 significantly reduced the expression of *CD44*, *Lgr5* and *BMI-1*. An enhancement of stemness by SCF was blocked by ectopic expression of miR-34a. Taken together, our results show that the regulation of c-Kit by miR-34 may critically contribute to the tumor suppressive effects of miR-34, and therefore p53, in CRC and other tumor entities.

## RESULTS

### c-Kit is regulated in a p53-dependent manner in colorectal cancer cell lines

**Figure 1:**
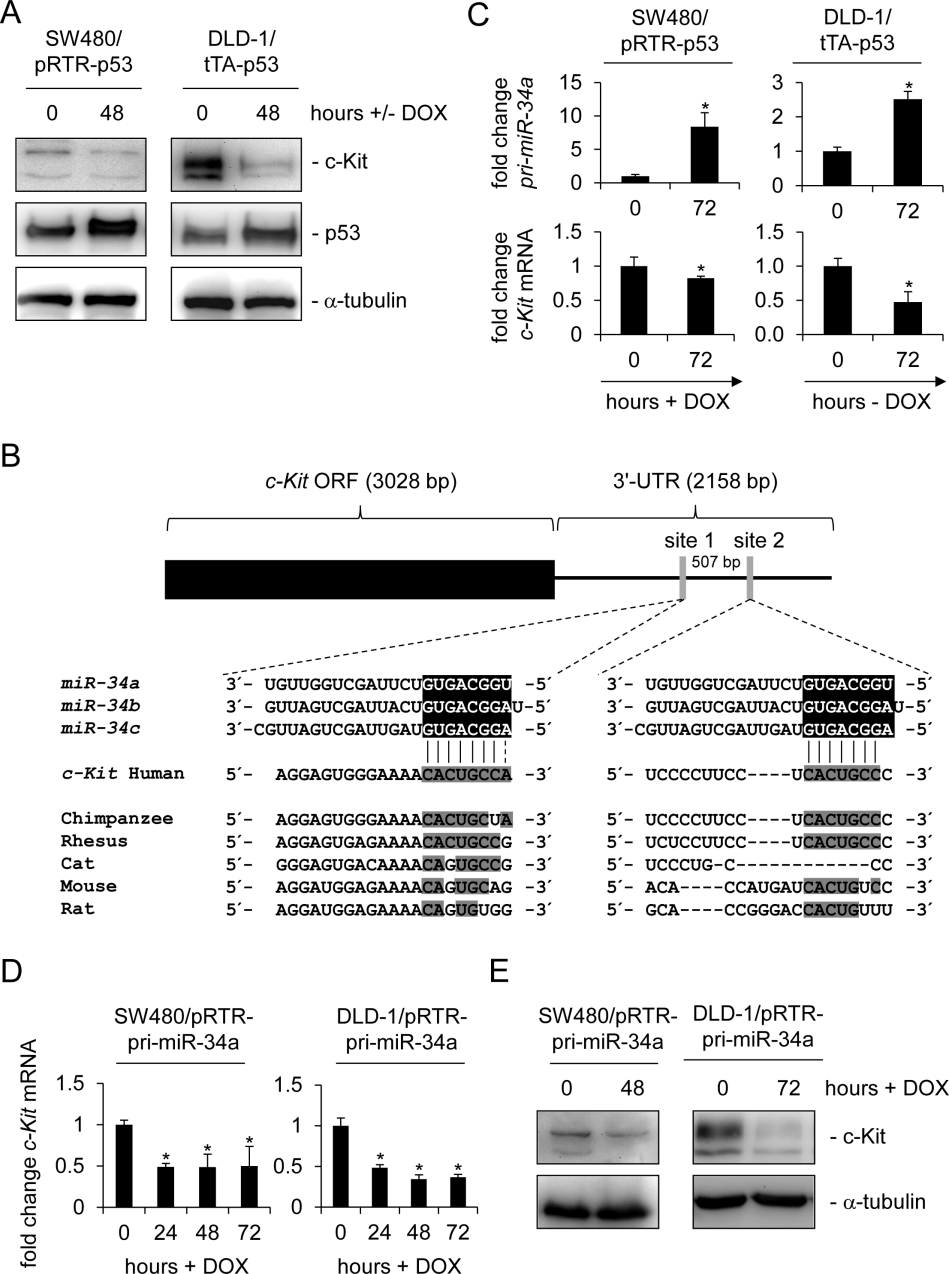
c-Kit is repressed after ectopic p53 and miR-34a expression in colorectal cancer cell lines (A) Western blot analysis of c-Kit protein levels after induction of p53 in colorectal cancer cell lines SW480 and DLD-1. α-tubulin served as a loading control. (B) Scheme of the *c-Kit* mRNA and conservation of the putative miR-34 seed-matching sequences, which are represented as grey vertical bars. Detailed sequences of the two sites and phylogenetic homologies are shown below. Potential base pairing is shaded in grey. (C) qPCR analysis of *pri-miR-34a* and *c-Kit* mRNA levels in the colorectal cancer cell lines SW480 and DLD-1 after induction of p53 by addition or withdrawal of doxycycline (DOX) for 72 hours. Results were normalized to *β-actin* mRNA. (D) qPCR analysis of *c-Kit* mRNA in the colorectal cancer cell lines SW480 and DLD-1 carrying the inducible pRTR/pri-miR-34a vector after addition of doxycycline. E) Detection of c-Kit protein by Western blot analysis after induction of *pri-miR-34a* in the indicated cells. α-tubulin served as a loading control. C+D: results represent the mean +/−S.D. (n=3).

**Figure 2:**
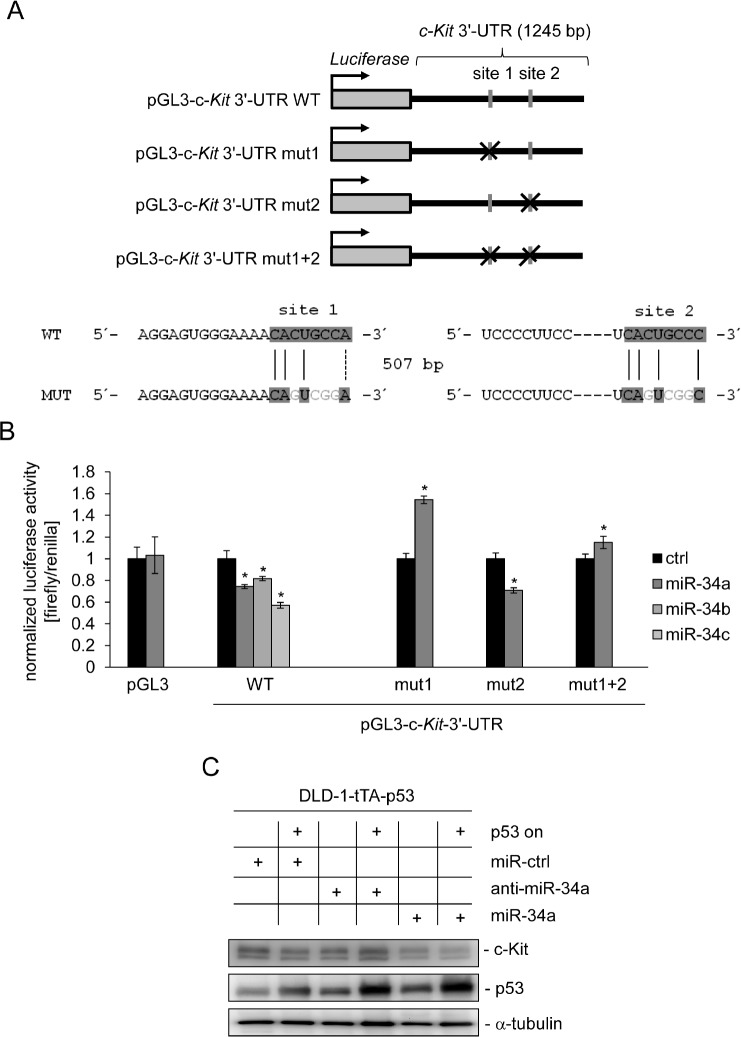
miR-34a directly targets c-Kit and mediates c-Kit repression by p53 (A) Scheme of constructs used for dual luciferase assays. The positions of the putative miR-34 seed-matching sequences in the *c-Kit* 3'-UTR are depicted as a grey vertical bars, their mutations as crosses. Sequences of the respective targeted mutations are given below. (B) Dual reporter assay in SW480 cells transfected with miR-34a/b/c mimics or control oligonucleotide and the indicated 3'-UTR-reporter constructs for the human c-*Kit* 3'-UTR. Data are represented as mean ± SD (n = 3). (C) DLD-1/tTA-p53 cells were either transfected with a control oligonucleotide, miR-34a or an antagomiR directed against miR-34a for 24 hours either in the presence or absence of DOX (without or with ectopic p53). Expression of the indicated proteins was detected by Western blot analysis. α-tubulin served as a loading control.

Since a recent study reported a p53-dependent regulation of c-Kit, which occurred in the absence of p53 binding to the c-*Kit* promoter in mice [32], we hypothesized that miR-34 could be the mediator of this effect. In order to investigate this putative connection we used two different systems to conditionally express p53: SW480 cell pools transfected with the doxycycline (DOX) -inducible vector pRTR expressing the *p53* open reading frame (ORF) and a DLD-1 single cell clone harboring a *p53* allele under control of the tet-off system [36, 37]. Although the endogenous levels of c-Kit were lower in SW480 cells than in DLD-1 cells, activation of p53 in both cellular systems resulted in the down-regulation of c-Kit protein expression (Figure 1A). Since miRNAs were shown to mediate gene repression by p53 we examined the c-*Kit* 3'-UTR using the Target-Scan algorithm [38]. Thereby we identified two potential miR-34 seed-matching sequences in the 3'-UTR of c-*Kit* (Figure 1B). While the first site (which is a perfect match to the miR-34a 8-mer seed-matching sequence) is relatively conserved among different species, the second site seems to be less conserved. In line with previous reports, expression of the primary *miR-34a* transcript was induced and the c-*Kit* mRNA was repressed after p53 activation in both SW480 and DLD-1 cells (Figure 1C). Since the expression of miR-34b and miR-34c is at least 100 fold lower than that of miR-34a [39-41] in CRC cells and cell lines we focused our further studies on miR-34a. Notably, the ectopic expression of miR-34a driven by a conditional, episomal vector was sufficient to reduce c-Kit expression at the mRNA and protein levels in SW480 and DLD-1 cells (Figure 1D and 1E). Similar results were obtained with the CRC cell line HCT15 harboring the same miR-34 expression vector, though miR-34a mediated regulation was not as pronounced as in the other two cell lines (Supplemental Figure 1A and B). In order to determine whether miR-34 directly binds to the seed-matching sequences mentioned above we placed the c-*Kit* 3'-UTR (including the two potential binding sites) downstream of a luciferase open reading frame (Figure 2A). In a dual-reporter luciferase assay miR-34a as well as miR-34b and c significantly decreased the activity of this reporter (Figure 2B). When the seed-matching sequence in site 1 was mutated, the reporter was resistant to down-regulation by miR-34a, whereas mutation of site 2 did not affect the miR-34-mediated down-regulation of the reporter. Mutation of both sites led to resistance against miR-34a regulation. These results indicate that the miR-34 family directly targets the c-*Kit* 3'-UTR via site 1. This result is in accordance with the higher degree of conservation of site 1 when compared to site 2. Furthermore, p53-mediated down-regulation of c-Kit in DLD-1 cells could be prevented by simultaneous transfection of an antagomiR directed against miR-34a (anti-miR-34a), while additionally transfected miR-34a further enhanced the repression of c-Kit when p53 was activated concomitantly (Figure 2C). Taken together, miR-34a therefore mediates the repressive effects of p53 on c-Kit expression by directly targeting the c-*Kit* 3'-UTR via a single conserved seed-matching sequence.

### miR-34a inhibits Erk signaling and colony formation by down-regulation of c-Kit

**Figure 3:**
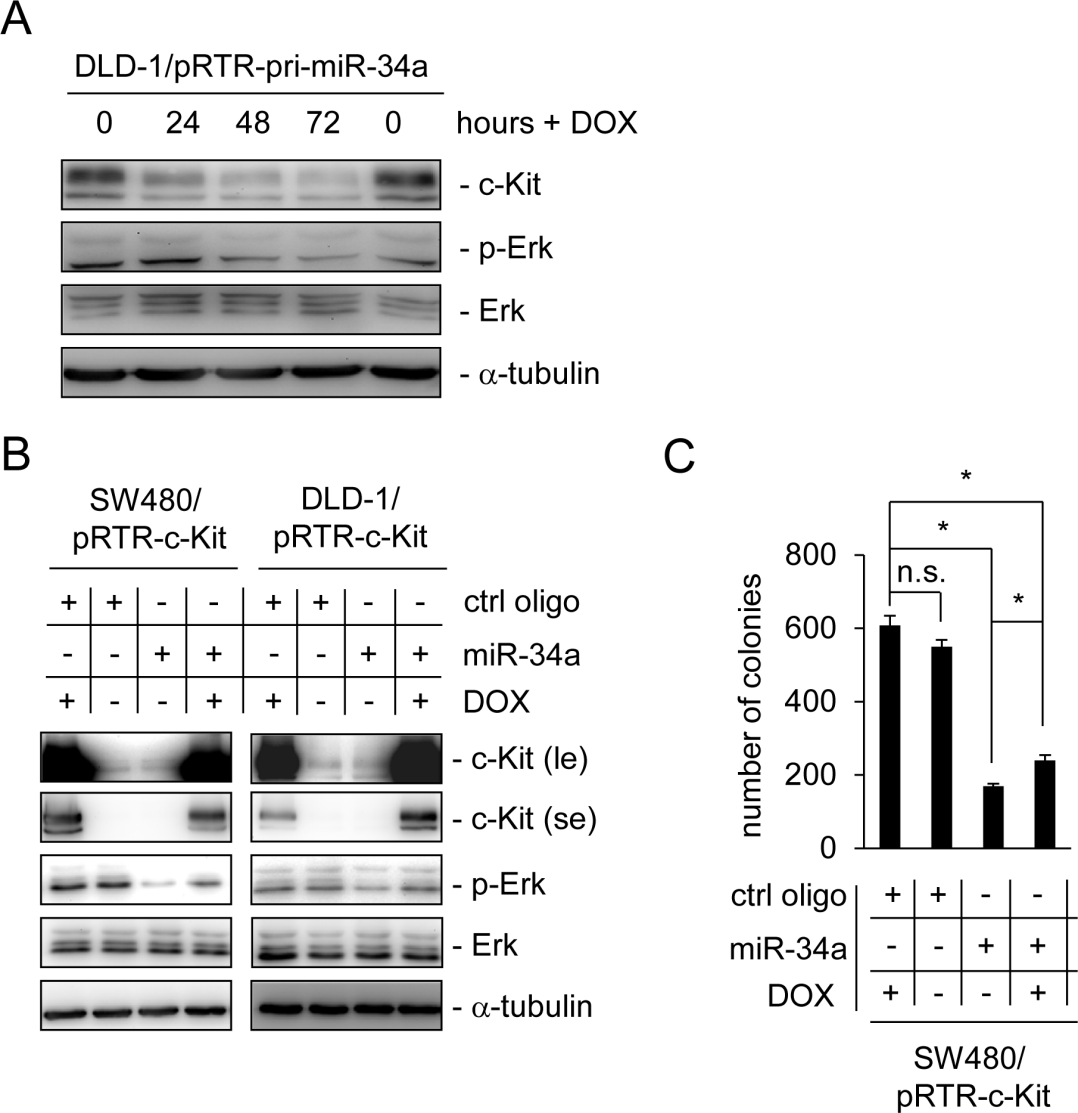
Ectopic c-Kit overrides miR-34a-dependent inhibition of Erk signaling and colony formation in soft agar (A) DLD-1/pRTR-pri-miR-34a cells were treated with DOX for the indicated time-points. The indicated proteins were detected by Western blot analysis. α-tubulin served as a loading control. (B) Western blot analysis of the indicated SW480 and DLD-1 cells after oligonucleotide transfection for 48 hours and addition of DOX for 24 hours. α-tubulin served as a loading control. le = long exposure, se = short exposure. (C) Soft agar colony formation assay. The indicated cells were treated as described under (B). Results represent the mean +/−S.D. (n=3) and significance was calculated applying a Student's t-test.” * “: p < 0.05.

In order to determine the consequences of a miR-34a-mediated decrease in c-Kit protein we analyzed Erk signaling, which is known to be activated by c-Kit. Indeed, the levels of phosphorylated Erk were decreased following the induction of ectopic miR-34a expression in DLD-1 cells, which was accompanied by a reduction in the levels of c-Kit protein, whereas the total amount of Erk protein levels was not affected (Figure 3A). Next, we determined whether the miR-34a-mediated decrease in Erk-phosphorylation was dependent on the down-regulation of c-Kit. Therefore, we generated pools of DLD-1 and SW480 cells carrying a conditional expression vector for c*-Kit* lacking its original 3'-UTR and therefore a miR-34 seed matching sequence. Ectopic miR-34a expression resulted in decreased levels of Erk-phosphorylation in both cell lines, while the control oligo had no effect (Figure 3B). Notably, induction of ectopic c-Kit largely reversed the effect of miR-34a on phosphorylated Erk, whereas the amount of total Erk was not affected. Collectively, these results show that the down-regulation of c-Kit is necessary for the inhibitory effects of miR-34a on mitogenic Erk signaling.

To test whether these regulations would affect the capacity of SW480 cells to grow in soft agar, which is known to be affected by Erk-signaling [42], cells treated as in Figure 3B were seeded into soft agar and colony formation was measured (Figure 3C). Indeed, miR-34a transfection severely reduced the number of colonies. Furthermore, induction of ectopic c-Kit expression led to a slight but significant increase in the number of colonies, at least partially reflecting the results observed on the level of Erk phosphorylation. Therefore, signaling downstream of c-Kit contributes to colony formation and can be suppressed by miR-34a via down-regulation of the c-Kit receptor expression. We also analyzed the expression of the EMT marker genes *E-cadherin* and *Vimentin* as well as the primary transcript of *miR-34a* upon ectopic c-Kit expression in DLD-1 and SW480 cells (Supplemental Figure 1C and D). We did not detect any significant changes of these mRNAs, although recent publications implied a role of c-Kit in EMT [43, 44].

### The interplay between miR-34a and c-Kit influences chemo-sensitivity

**Figure 4:**
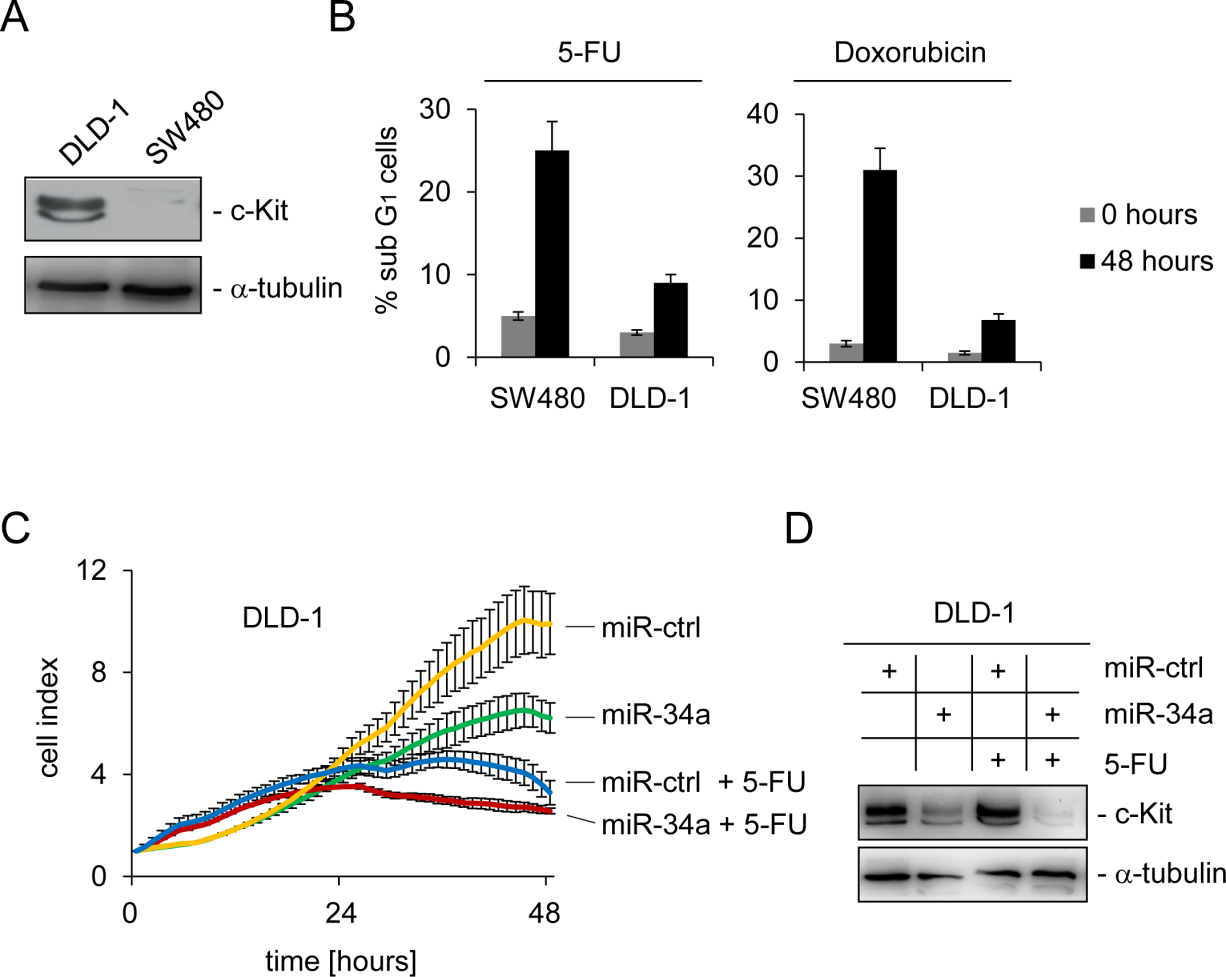
c-Kit and miR-34a modulate the apoptotic response to chemotherapeutic agents (A) Detection of endogenous c-Kit levels by Western blot analysis of DLD-1 and SW480 colorectal cancer cells. Detection of α-tubulin served as loading control. (B) Cells were treated with either 5-FU or Doxorubicin for 48 hours and subjected to DNA content analysis by flow cytometry. Results represent the mean +/−S.D. (n=3). (C) DLD-1 cells were transfected with the indicated oligonucleotides and simultaneously treated with 5-FU or water. The cell index, which corresponds to cell proliferation, was determined by real-time impedance measurements. (D) Cells treated as described in (C) were subjected to Western blot analysis. α-tubulin served as loading control.

Recently, c-Kit was shown to mediate chemo-resistance in ovarian tumor initiating cells [19]. Therefore, we asked whether c-Kit might play a similar role in colorectal cancer. To address this issue DLD-1 cells, which express high levels of endogenous c-Kit, and SW480 cells expressing low levels of c-Kit were compared (Figure 4A). After treatment of both cell lines with either the anti-metabolite 5-FU or the anthracycline antibiotic Doxorubicin for 48 hours, SW480 cells showed a more pronounced apoptotic response to the treatment with both chemo-therapeutics, as determined by DNA content analysis using flow-cytometry, than DLD-1 cells (Figure 4B): upon treatment with the 5-FU the apoptotic sub-G_1_ fraction of cells was elevated about five times (from five to 25 %) in SW480 cells, while it tripled in DLD-1 cells (from three to nine %). Similar results were observed with Doxorubicin treatment: while SW480 cells showed a 10 fold increase (from three to 31 %) of apoptosis, apoptosis in DLD-1 cells increased about 3.5 fold (2 to 7 %). Consistently, the rate of spontaneous apoptosis in untreated cells was higher in SW480 cells than in DLD-1 cells, which therefore also inversely correlated with the level of c-Kit expression.

In order to determine whether the relative chemo-resistance of DLD-1 cells is based on high levels of c-Kit and can therefore be influenced by miR-34a, DLD-1 cells were transfected with miR-34a or control oligos for 24 hours to achieve c-Kit down-regulation. Thereafter, cells were treated with either 5-FU or left untreated and the cell index was measured continuously over two days by real-time impedance measurements. Cells transfected with the control oligo appeared unaffected and transfection with miR-34a resulted in a lower cell index over time (Figure 4C). Treatment with 5-FU affected the survival of cells even stronger but the most drastic effect resulted from the combined treatment with miR-34a and 5-FU. As expected, ectopic miR-34a resulted in a severe decrease of c-Kit protein levels in DLD-1 cells, whereas the control oligonucleotide did not influence expression of c-Kit protein (Figure 4D). Taken together, these results show that high levels of c-Kit promote chemo-resistance of CRC cells to 5-FU and Doxorubicin, respectively. This is probably due to reduction of the apoptotic response. miR-34a can inhibit this effect via down-regulation of c-Kit and therefore sensitize cells to chemotherapeutic treatment.

### Disruption of the SCF/c-Kit axis by miR-34a inhibits migration and invasion

**Figure 5:**
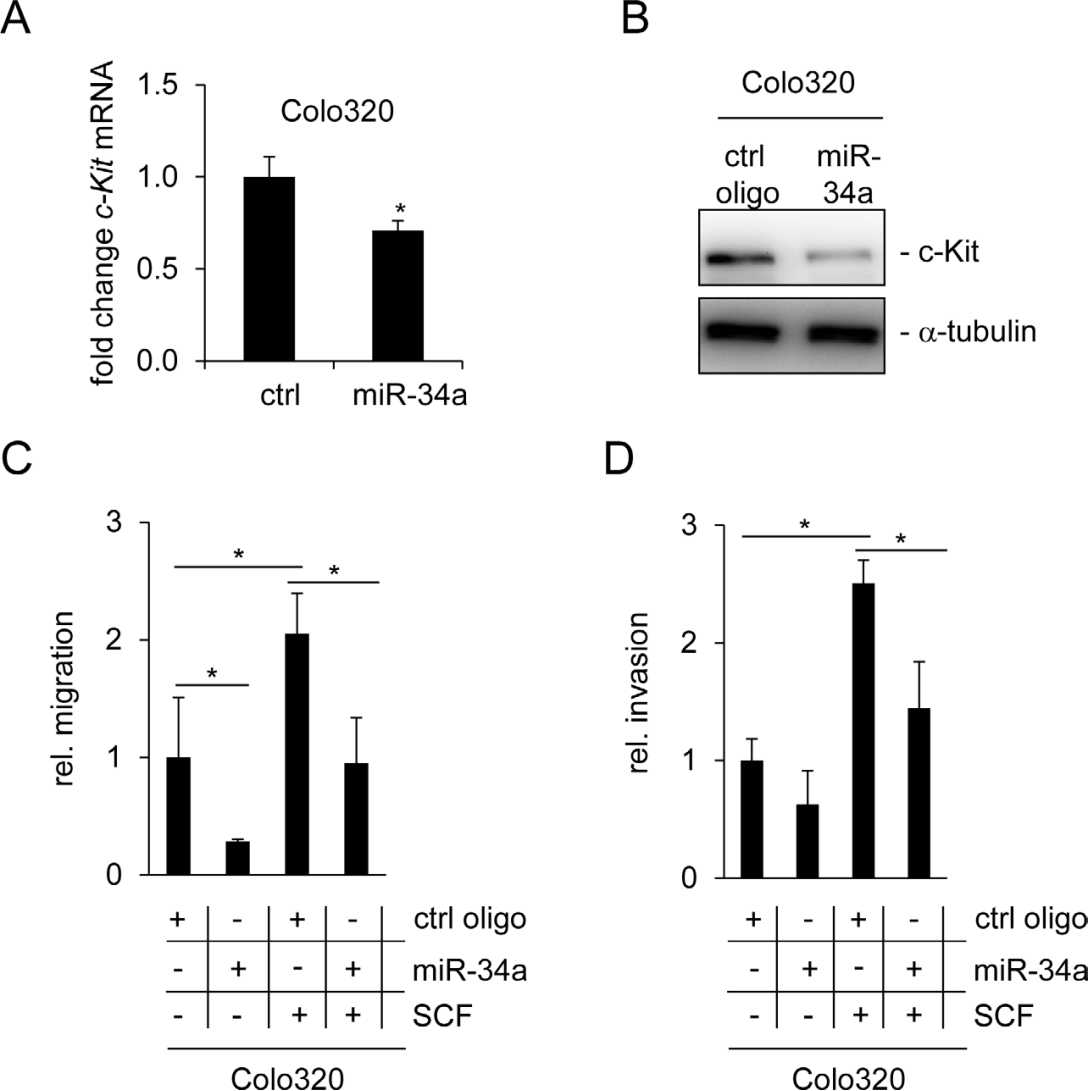
miR-34a inhibits basal and SCF-induced migration and invasion of Colo320 cells (A) Colo320 CRC cells were transfected either with a control (ctrl) or a miR-34a oligonucleotide for 48 hours. qPCR analysis was used to determine *c-Kit* mRNA levels. (B) Detection of c-Kit protein levels by Western blot analysis 48 hours after oligonucleotide transfection. α-tubulin served as loading control. (C) Colo320 cells were transfected with the indicated oligonucleotides and simultaneously treated with SCF (or water) for 24 hours. Thereafter cells were seeded into Boyden chambers to determine migration. (D) Determination of invasion using a Boyden chamber assay. Cells were treated as in (C). (A,C,D) Results represent the mean +/−S.D. (n=3) and significance was calculated applying a Student's t-test.” * “: p < 0.05.

Another function of the SCF/c-Kit axis is an enhancement of migration, which was previously observed after treatment of the CRC cell line Colo320 with SCF [45]. Therefore, we analyzed whether transfection of Colo320 cells with miR-34a may interfere with the effects of SCF. First, regulation of c-Kit by miR-34a was confirmed on mRNA and protein level (Figure 5A and B). As expected, transfection of Colo320 cells with miR-34a oligonucleotides for 48 hours led to a decrease of c-Kit on the mRNA and protein levels. As determined by a modified Boyden chamber assay, the ectopic expression of miR-34a resulted in a decrease in cell migration (Figure 5C). Conversely, when cells were treated with SCF, the number of migrating cells doubled, confirming results described by Yusada et al. [45]. Combined treatment of Colo320 cells with SCF and miR-34a mimetics significantly reduced migration approximately to the levels observed in the controls. miR-34a had similar inhibitory effects on basal invasion and SCF-induced invasion of Colo320 cells (Figure 5D). A likely explanation for these effects is that miR-34a interferes with SCF signaling by suppressing the expression of the c-Kit receptor which could render cells refractory to SCF.

Interestingly, we observed no change in the expression of EMT markers (*Vimentin*, *CDH1*, *Occludin* and *Fibronectin*) in Colo320 cells after ectopic miR-34a expression (Supplemental Figure 1E). This suggests that the underlying mechanism is not related to the induction of MET by miR-34a, which was previously shown to occur in CRC cells [36]. Also expression of *pri-miR-34a* was not affected by c-Kit activation, excluding the presence of a feed-back loop, which was recently described for miR-34 and its target Snail [36].

### Association of c-Kit with markers of CRC stemness

**Figure 6:**
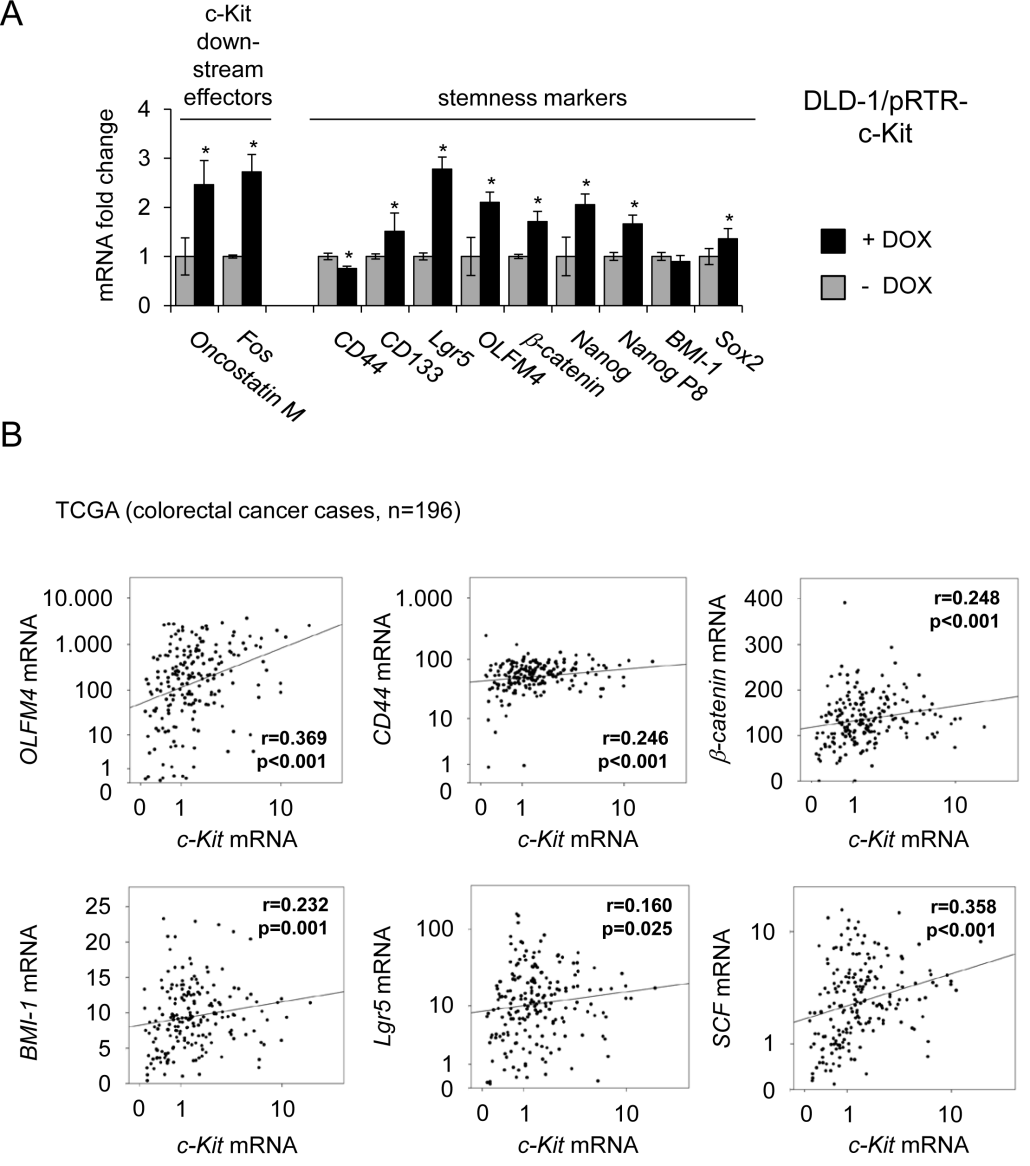
Ectopic c-Kit induces expression of stemness markers and correlates with their expression in primary CRC (A) DLD-1/pRTR-c-Kit cells were treated with DOX for 48 hours. Expression of the indicated mRNAs was determined by qPCR. Results represent the mean +/−S.D. (n=3).” * “: p < 0.05. (B) mRNA expression data derived from primary CRC samples (n = 196) were obtained from the public database TCGA [54]. Correlation coefficients and p-values were calculated applying the Spearman correlation algorithm. Scatter plots show the respective correlations. Both, the c-*Kit* levels on the x-axes and the y-axes of the *OLFM4, CD44, Lgr5* and *SCF* mRNA expression values are provided as log_10_ scale.

Since SCF is involved in the regulation of hematopoietic stem cells [46] and recent studies indicated a role for c-Kit in stemness in ovarian cancer [19], we asked whether c-Kit influences the expression of stemness markers in colorectal cancer cells. Therefore, c-Kit was ectopically expressed in DLD-1 cells and changes in mRNA expression were determined by qPCR. As positive controls for c-Kit-mediated gene regulations, expression of its downstream effectors c-*Fos* and *Oncostatin M* [47, 48], was analyzed. As markers for CRC stemness the expression of *CD44*, *CD133*, *Lgr5*, *Nanog*, *Nanog P8*, *β-catenin*, *Sox2*, *BMI-1* and *OLFM4* was determined [49-53]. As expected, *c-Fos* and *Oncostatin M* were induced after c-Kit activation (Figure 6A). Besides *CD44* and *BMI-1*, all analyzed stemness markers were significantly up-regulated by c-Kit. *CD44* was repressed and *BMI-1* remained unchanged. These results indicate that c-Kit might enhance stemness of colorectal cancer cells. Next, we determined whether an association between c-Kit and stemness markers may also be present in clinical samples obtained from CRC patients as well. Therefore, expression data of 196 colorectal tumors from the public database TCGA (The Cancer Genome Atlas, [54]) was analyzed (Figure 6B). From these analyses, the expression of *OLFM4, CD44, β-catenin, BMI-1* and *Lgr5* mRNA emerged as significantly associated with elevated c-*Kit* mRNA expression (p ≤ 0.05). Additionally, elevated c-*Kit ligand* (*SCF/stem cell factor*) expression correlated with high receptor expression. This confirms previous results describing a co-expression of receptor and ligand [22-25]. Collectively, these associations suggest that c-Kit or/and presumably factors regulating c-Kit, as miR-34, might play a role in the regulation of stemness in colorectal cancer.

### miR-34 interferes with SCF-induced stemness

**Figure 7:**
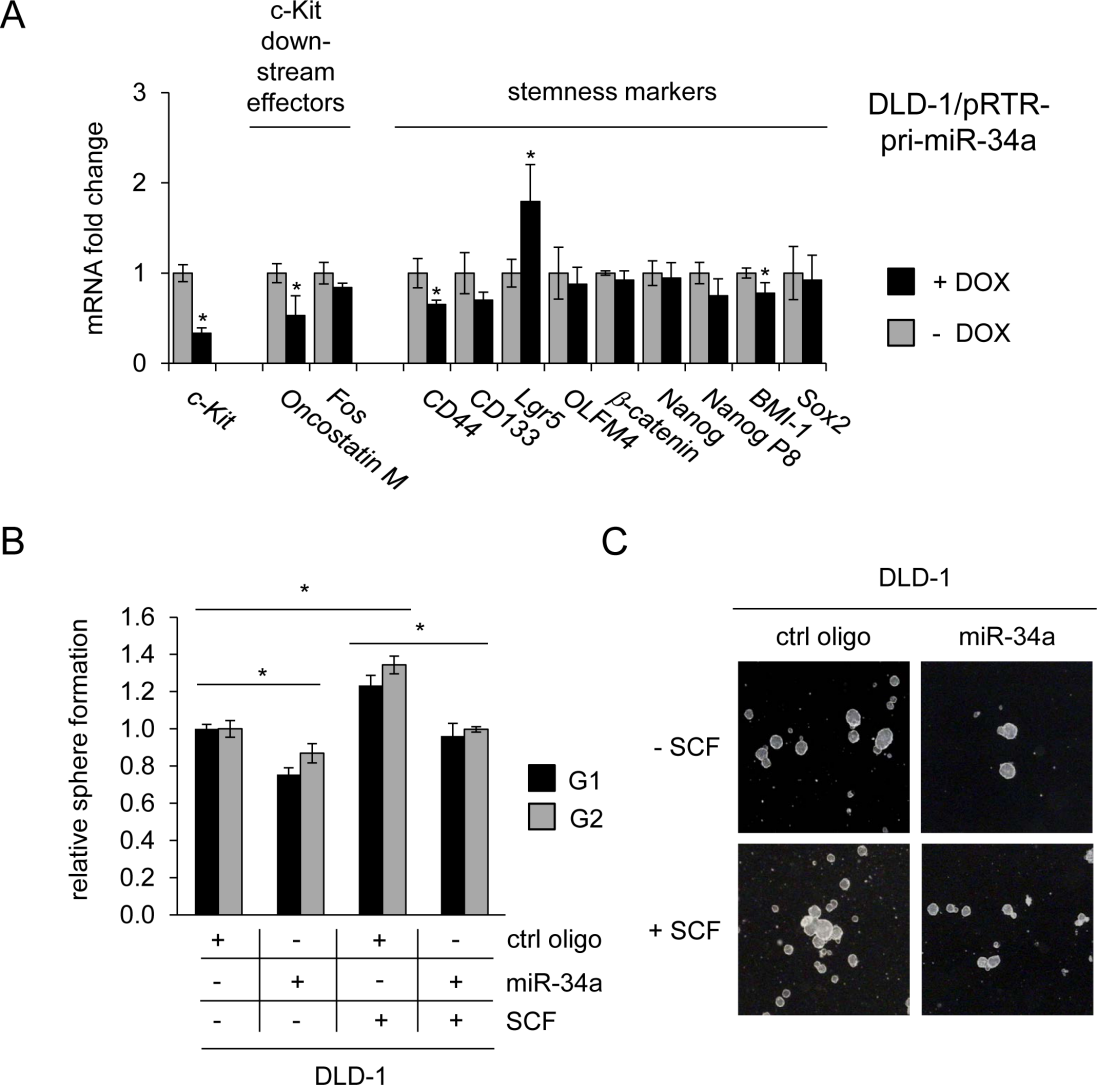
Role of the miR-34a/c-Kit axis in the regulation of stemness markers and sphere formation (A) DLD-1 cells pRTR/pri-miR-34a were treated with DOX for 48 hours. Expression of the indicated mRNAs was analyzed by qPCR. (B) DLD-1 cells were transfected with the indicated oligonucleotides and treated with SCF (or water) and subjected to a sphere formation assay. Sphere numbers were determined after seven days for the first generation (G1) and seven days after seeding for G2. Treatment with oligonucleotides and SCF was repeated when cells were passaged. (C) Representative pictures of DLD-1 derived G1 spheres, magnification: 40x. (A,B) Results represent the mean +/−S.D. (n=3) and significance was calculated applying a Student's t-test.” * “: p < 0.05.

To investigate whether miR-34a-dependent regulation of c-Kit might affect c-Kit-induced stemness markers, miR-34a was ectopically expressed in DLD-1 cells by activation of a conditional pRTR-pri-miR-34a vector (Figure 7A). The mRNA levels of c-*Kit* and its published downstream effectors *c-Fos* and *Oncostatin M* were repressed upon activation of miR-34a expression. However, mRNAs representing stemness markers displayed relatively weak down-regulations upon induction of miR-34a. In order to determine whether these regulations are relevant for stemness-related properties on the cellular level, DLD-1 cells were subjected to a sphere formation assay (Figure 7B). This assay in which cells grow in suspension under non-adherent conditions can be used to evaluate self-renewing capacities of cancer stem cells and has been applied previously for analyzing the effect of miR-34 on gastric cancer stem cells [55]. In line with previous publications [56] treatment with SCF significantly stimulated sphere-formation of DLD-1 cells. In contrast, treatment with miR-34a oligonucleotides reduced the number of spheres and also suppressed SCF-induced sphere formation of DLD-1 cells (Figure 7B and C). These effects were consistently observed over two consecutive generations. Taken together, these results show that miR-34a suppresses sphere formation and therefore stemness of colorectal cancer cells by targeting c-Kit.

## DISCUSSION

**Figure 8:**
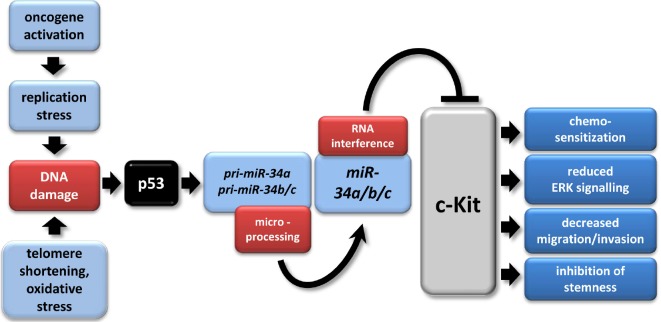
The p53/miR-34/c-Kit axis The model depicts multiple tumor suppressive effects of miR-34a directly targeting c-Kit which were identified in this study. In tumors, p53 mutation/inactivation or CpG methylation of miR-34a/b/c promoters may abrogate this pathway [33, 91, 92]. miR-34a/b/c have multiple other targets besides c-Kit [33, 93]. The model was adapted from [94].

In this study c-Kit was identified as a new target of the miR-34 family. Since c-Kit plays a role in numerous cancer-associated pathways, these results extend the tumor suppressive mechanisms of miR-34. A model summarizing our findings is shown in Figure 8. miR-34-mediated down-regulation of c-Kit resulted in diminished Erk-phosphorylation levels, which was associated with decreased colony formation in soft agar and therefore a decrease in cellular transformation. Moreover, low endogenous c-Kit protein levels or a decrease in c-Kit caused by ectopic miR-34 were associated with increased chemosensitivity. Furthermore, miR-34a inhibited migration and invasion and also SCF-mediated enhancement of these processes was blocked by miR-34a. Finally, activation of c-Kit induced several stemness markers in CRC cell lines and was associated with their expression in primary CRC tumors, whereas activation of miR-34a in CRC cell lines resulted in a down-regulation of c-Kit and c-Kit-induced markers, and suppressed sphere-formation of CRC cells, indicating that CRC stemness may be controlled by the miR-34/c-Kit axis.

Our studies were mainly carried out in colorectal cancer cells, which have been previously shown to express c-Kit [17, 45]. However, the role of c-Kit in patients with colorectal cancer is unclear and former studies described divergent results: Friedrichs et al found elevated c-Kit expression to be rare (17.1%) in colorectal carcinomas [57], which was in accordance with other studies which found c-Kit to be expressed at very low levels in colorectal cancer samples using different techniques [58, 59]. Further studies found c-Kit expression in 59% of stage II colorectal cancer patients [60], in 90% of normal colon mucosa and 30% of neoplastic tissues [61] and in 15% of primary colorectal tumors and 14% of distant metastases [62]. Though these results in patients are not uniform, c-Kit presumably has a function in colorectal cancer cells since the c-Kit receptor as well as its ligand are expressed at elevated levels in colorectal cancer cell lines [63]. In support of this, SCF stimulates anchorage independent growth in four out of five CRC cell lines [56] and increases migration of CRC cell lines [45]. Furthermore, aberrant activation of the c-Kit axis suppresses apoptosis and stimulates invasion of DLD-1 colorectal cancer cells [17].

We could show that miR-34a antagonizes SCF, presumably by targeting c-Kit and therefore interrupting the signaling pathways down-stream of c-Kit which are responsible for the changes in cell behavior. Noteworthy, ectopic c-Kit did not affect the expression of EMT markers, such as *CDH1/E-cadherin* or *Vimentin*. This was surprising, since previous publications have linked c-Kit with EMT, which is an important step in the metastatic cascade [43].

Our results indicate that c-Kit expression is associated with chemo-resistance, which can be partially reverted by ectopic miR-34a. This is reminiscent of the effect of Imatinib (STI571), which also targets c-Kit and was shown to be therapeutically effective against gastrointestinal stromal tumors/GIST [64, 65]. Imatinib renders DLD-1 cells, which express high c-Kit levels, more responsive to the cytotoxic effect of 5-FU [66]. Therefore, the additive effect of c-Kit inhibition and 5-FU has precedence and should be studied further, since this would potentially allow to reduce the dose of chemotherapeutic compounds in the future. miR-34 mimetics were previously shown to sensitize cells belonging to other tumor entities, such as breast, prostate, gastric and lung cancer towards chemotherapy [67-70]. Therefore, miR-34 mimetics represent promising candidates for clinical applications [71].

Furthermore, we found that c-Kit activation results in the up-regulation of several stemness markers in colorectal cancer cells and primary tumors. This is in accordance with previous findings, which show that colorectal cancer stem cells display increased resistance towards chemotherapeutics such as 5-FU [72, 73]. c-Kit was suggested to activate ABCG2 drug transporters to confer chemoresistance in ovarian cancer [19] which might explain why stem cells are resistant to a variety of agents, rather than to one single chemotherapeutic agent [49].

Interestingly, previous publications revealed that miR-221 and 222 regulate angiogenic processes via targeting c-Kit [74]. Since miR-34a targets c-Kit as well, effects of p53 on tumor-angiogenesis may be mediated, at least in part, via miR-34.

Recently, other RTKs were identified as targets of miR-34, for example Axl, c-Met, PDGFR α and β [49, 55, 75, 76], suggesting a coordinated inhibition of RTKs by miR-34. According to the microRNA target prediction algorithms targetSCAN and Miranda [72, 73], most of the potential miR-34-regulated RTKs cluster in class III [50], among them PDGFR α, PDGFR β and c-Kit.

Since both, miR-34a and c-Kit interact with numerous signaling pathways, more detailed analyses are necessary in order to illuminate the different aspects and consequences of this regulation. For instance activation of c-Kit is not only associated with cancer but seems to play a role in allergic asthma as well [6]. Recently, miR-34a was shown to interfere with mTOR signaling pathway and thereby to play a role in mast cell survival – a process critical in allergic disorders [77]. Moreover, TGF-β was reported to suppress miR-34 thereby leading to secretion of the chemokine CCL22, which is a direct target of miR-34 and recruitment of regulatory T cells [78]. These findings place miR-34 in the context of allergic disorders and miR-34-dependent down-regulation of c-Kit might therefore be important for understanding and treating these diseases as well. Interestingly, inhibition of different RTKs and particularly c-Kit seems to affect asthma [79, 80], via decreasing histamine levels, infiltration of mast cells and eosinophils, interleukin-4 production and airway hyper-responsiveness [81].

Replacement therapy with miR-34a mimetics was successful in several preclinical studies using mouse models of cancer and may therefore represent a therapeutic option for the treatment of several tumor entities in the future [82-86]. Our results indicate that c-Kit may be up-regulated in tumors due to inactivation of miR-34 either by CpG methylation or p53 mutations/inhibition. Such tumors may be especially sensitive to a replacement of miR-34 using mimetics.

## MATERIALS AND METHODS

### Cell culture and treatments

The colorectal cancer cell lines SW480 and DLD-1 were kept in DMEM and McCoy's medium, respectively. Colo320 and HCT15 cells were kept in RPMI medium. If not indicated differently, all media were supplemented with 10% FBS (Invitrogen). All cells were cultivated in the presence of 100 U/ml penicillin and 0.1 mg/ml streptomycin. Cells were transfected with the episomal expression vector pRTR [87] using Fugene6 (Roche). Polyclonal cell pools were generated by selection with puromycin (2 μg/ml) for 10 days. The percentage of GFP-positive cells was determined 72 hours after addition of doxycycline (DOX) at a final concentration 100 ng/ml. Transfection of cells with antagomiRs, pre-miRNAs/mimics and respective negative controls (Ambion) was performed using HiPerfect (Qiagen) at a final oligonucleotide concentration of 50 nM. Treatment of cells with 5-Fluorouracil (5-FU, Sigma-Aldrich) was conducted at a concentration of 20 μg/ml, while Doxorubicin (Sigma-Aldrich) was used at a concentration of 0.25 μg/ml. 5-FU, a pyrimidine analog and anti-metabolite [88] trademarked as Efudex, and the anthracycline antibiotic Doxorubicin [89] are often used for chemotherapy. SCF (ImmunoTools) was used at a final concentration of 10 ng/ml.

### Western Blot Analysis

SDS-PAGE and Western blotting were performed according to standard protocols. Cells were lysed in RIPA lysis buffer (50 mM Tris/HCl, pH 8.0, 250 mM NaCl, 1% NP40, 0.5% (w/v) sodium deoxycholate, 0.1% sodium dodecylsulfate, complete mini protease inhibitors (Roche) and PhosSTOP Phosphatase Inhibitor Cocktail Tablets (Roche)). Lysates were sonicated and centrifuged at 16.060 g for 20 min at 4°C. Per lane 60-100 μg of whole cell lysate was separated using 7.5% SDS-acrylamide gels and transferred on Immobilon PVDF membranes (Millipore). ECL signals were recorded using a CF440 Imager (Kodak). Antibodies used for detection of the indicated proteins are shown in Supplemental Table 1.

### Generation of a c-*Kit* expression vector

The vector pWPTet containing the coding sequence of human c-*Kit* was kindly provided by Matthias Mayerhofer. The coding sequence was excised using BamHI and SpeI, cloned into the shuttle vector pUC19SfiI and ligated into pRTR, respectively via the SfiI sites. The sequence was verified by sequencing. The generation of pRTR vectors containing p53 and pri-miR-34a was described previously [52]. A list of all plasmids used in this paper is provided in Supplemental Table 2.

### Flow cytometric analysis of DNA content/apoptosis and GFP/RFP expression

Cells were seeded in 6-well plates (2×10^5^ cells/well) and cultured in the presence or absence of 100 ng/ml DOX. For flow cytometry, cells were trypsinized after 72 hours for GFP/RFP analysis, washed and resuspended in PBS. For detection of GFP expression 10,000 cells per sample were analyzed with a C6 Flow Cytometer Instrument (BD Accuri). For DNA content analysis (1×10^5^) SW480 cells were seeded per 6-well and transfected with 50nM of miR-34a or control oligo. After 24 hours cells were treated with DOX (final concentration 100 ng/ml [Sigma]) or water and incubated for another 48 hours until harvesting. Floating cells and trypsinized cells were collected by centrifugation at 1200 rpm (600 g) for 5 minutes, fixed with ice-cold 70% ethanol and stored over night on ice. After washing with phosphate-buffered saline (PBS), 0.5 ml FACS solution (PBS, 0.1% Triton X-100, 60 mg/ml propidium iodide (PI) and 0.5 mg/ml RNase) was added per sample and incubated at 37°C for 30 min. DNA content was determined analyzing 10,000 cells per sample using a C6 device (Accuri, Erembodegem, Belgium) and three independent samples per treatment.

### Cloning of the c-*Kit* 3'-UTR

The 3'-UTR of c-*Kit* mRNA containing putative *miR-34a* binding sites was PCR-amplified from oligo-dT-primed cDNA from human diploid fibroblast cells with the Verso cDNA kit (Thermo Scientific). The 3'-UTR was cloned into pGL3-control-MCS [90] and verified by sequencing. Mutagenesis of seed sequences was done with the QuickChange Mutagenesis Kit (Stratagene) according to manufacturer's instructions. Oligonucleotides used for cloning and mutagenesis are provided in Supplemental Table 3.

### Dual Luciferase Reporter Assays

SW480 cells were seeded in 12-well format at 1×10^4^ cells/well, and transfected after 48 hours with 100 ng of the indicated firefly luciferase reporter plasmid, 20 ng of *Renilla* reporter plasmid for normalization and 25 nM of *miR-34a/b/c* pre-miRNA (Ambion, PM11030) or a negative control oligonucleotide (Ambion, neg. control #1). Luciferase assays were carried out after 48 hours with the Dual Luciferase Reporter assay system (Promega) according to manufacturer's instructions. Fluorescence intensities were measured with a luminometer (Berthold) in 96-well format and analyzed with the Simplicity software package (DLR).

### Quantitative Real-Time PCR

Total RNA was prepared with the High Pure RNA Isolation Kit (Roche) according to manufacturer's instructions. cDNA was generated from 1 μg of total RNA per sample using anchored oligo-dT primers (Verso Kit, Thermo Fischer). All results were normalized to *β-actin*. Per time point/condition three independent experiments were conducted. RT-qPCR was performed using the LightCycler (Roche) and the Fast SYBR Green Master Mix (Applied Biosystems). A list of qPCR-primers used for this study is provided in Supplemental Table 4.

### Soft agar colony formation assay for anchorage-independent growth

The bottom of a 12-well was coated with 700 μl base agar containing 0.8% low melt agarose (Lonza) which was then covered with 700 μl 0.4% agarose containing 2,000 SW480 cells, either transfected with miR-34a or control oligo 24 hours before, and incubated for 24 hours at 37°C and 5% CO_2_. 24 hours later, 250 μl medium supplemented with 10% FBS was added supplemented with either doxycycline (final concentration 100 ng/ml [Sigma]; stock solution 100 μg/ml in water) or vehicle (water). Cells were incubated for 14 days changing the media every 3 days. For determination of colony numbers cells were stained with 500 μl of 0.005% crystal violet per well for 2 hours and de-stained in PBS over night at 4°C. Pictures were taken using an EOS 400D camera (Canon) and colonies were counted using image J software (NIH).

### Real-time impedance measurement

A real-time cell analyzer (RTCA) (xCelligence RTCA SP; Roche Diagnostics GmbH, Penzberg, Germany) was used to assess cellular impedance according to manufacturer's instructions. 5000 cells were seeded into each well of an E-plate 16. The seeded cells were allowed to equilibrate for at least 30 minutes in the tissue culture incubator, then transfected or treated with doxycycline respectively before electrode resistance/impedance was recorded every 60 minutes. After 24 hours 5-FU was administered. The measurement was done every 60 minutes for 48 hours. The electrical impedance is represented as a dimension/unit-less parameter termed cell-index, which represents the relative change in electrical impedance that occurs in the presence and absence of cells in the wells. This change is calculated based on the following formula: CI = (*Z*i −*Z*0)/15, where *Z*i determines the impedance at an individual experimental time point and *Z*0 is the impedance measured at the beginning of the experiment. The impedance is measured at three different frequencies (10, 25 or 50 kHz) (ref: Roche Diagnostics GmbH. Introduction of the RTCA DP Instrument. RTCA DP Instrument Operator's Manual, A. Acea Biosciences, Inc.; 2008.).

### Migration and invasion assays in Boyden chambers

In order to investigate the changes in motility Colo320 cells were seeded into six-well plates and immediately transfected with either miR-34a or a control oligo [100 nM each] and treated with SCF [10 ng/ml] or vehicle. After 24 hours cells were seeded into Boyden chambers (Corning) either coated (invasion) or uncoated (migration) with Matrigel (BD Bioscience) at a dilution of 3.3 ng/ml in medium without serum. Medium was supplemented with SCF for stimulated cases. Cells were allowed to migrate for 48 hours, then non-motile cells at the top of the filter were removed and the cells in the bottom chamber were fixed with methanol and stained with DAPI. Per membrane, the cell number was determined in five fields by fluorescence microscopy.

### Sphere formation assay

For induction of sphere formation adherent DLD-1 cells were trypsinized and 1 × 10^5^ cells were seeded into a six-well coated with attachment preventing PolyHEMA (Sigma) using 5 ml sphere-medium consisting of DMEM-F12 + GlutaMAX-I (Invitrogen) supplemented with B27 supplement (1:50; Invitrogen), EGF (20 ng/ml; R&D Systems), BSA (0.4 %, Sigma) and insulin (4 μg/ml; Invitrogen). [75]. Immediately after seeding the cells were transfected with either a control oligo or miR-34a [200 nM] and treated with either water or SCF [10ng/ml]. Resulting spheres were trypsinized again and quantified as well as employed for a second generation. For quantification 1 × 10^4^ cells/well were seeded in sphere-medium containing 1 % methyl cellulose (Sigma) into PolyHEMA coated 96-well plates (at least six wells per unicate). The number of colonies larger than 50 μm in diameter was determined after seven days. Representative pictures were taken using an EOS 400D camera (Canon) at 40 fold magnification.

### Statistical analysis

For analysis of patient data calculations were conducted using SPSS software 19 (SPSS Inc.). For the comparison of the expression data the Spearman correlation algorithm was applied for the generation of the correlation coefficient (r) and statistical significance (p-value). Correlations were illustrated by generating scatter/dot plots using the SPSS chart builder tool. A partial regression line was added in order to emphasize the respective correlation. For most assays a Student's t-test was applied to determine significance if not indicated otherwise. P-values of less than 0.05 were considered statistically significant.

## Supplementary Figure and Tables




