# Supplementary material for: Repression of c-Kit by p53 is mediated by miR-34 and is associated with reduced chemoresistance, migration and stemness
Source: Oncotarget. 2013 Aug 6;4(9):1399–415. doi: 10.18632/oncotarget.1202 (PMC3824539; doi:10.18632/oncotarget.1202)
Supplement: Supplementary file 1 [file oncotarget-04-1399-s001.pdf]

Repression of c-Kit by p53 is mediated by miR-34 and is associated with reduced chemoresistance, migration and stemness – Siemens et al

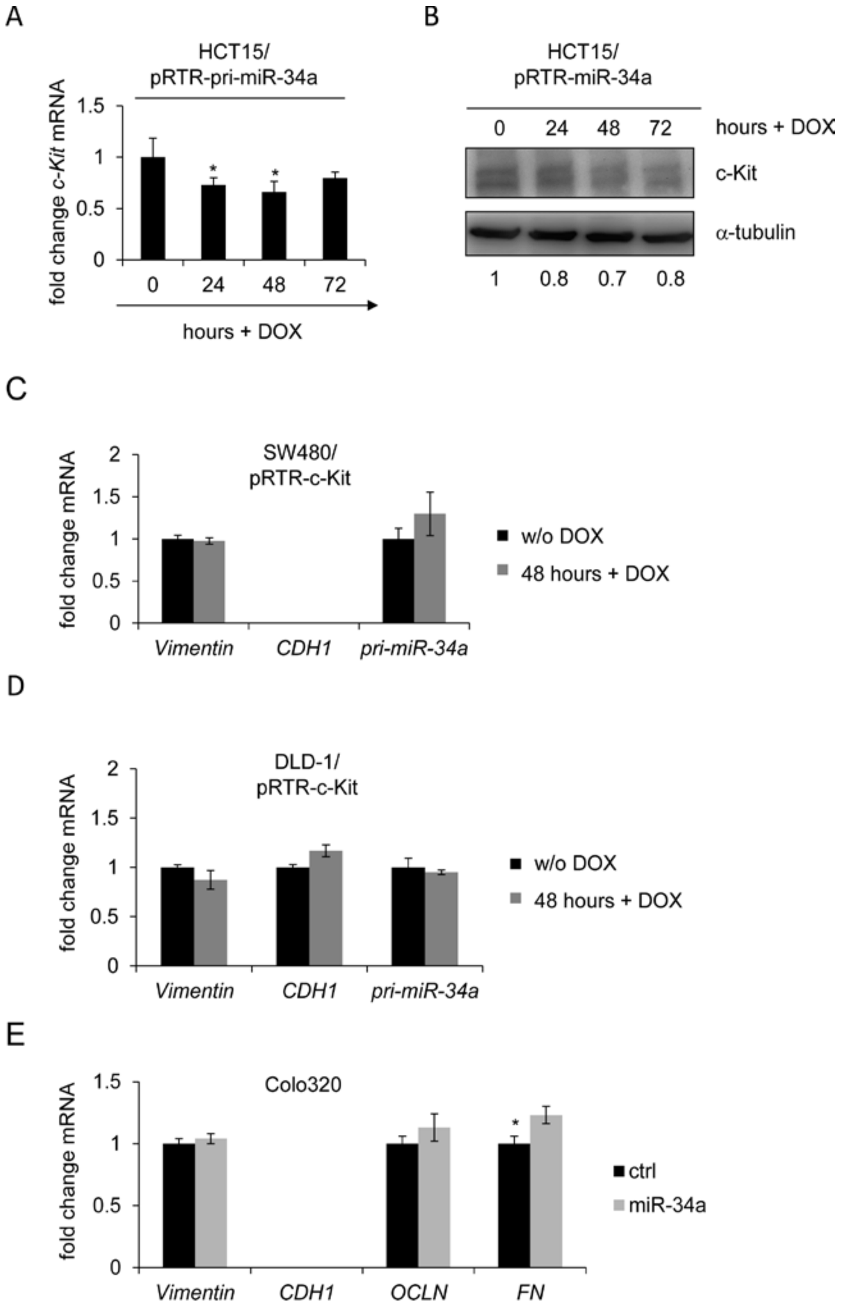

Supplemental Figure 1: (A) Measurement of c-*Kit* mRNA by qPCR upon addition of DOX to the colorectal cancer cell line HCT15/pRTR-pri-miR-34a vector. Results represent the mean +/-S.D. (n=3). ” * “ : $p < 0.05$. (B) Western blot analysis of c-Kit protein levels upon induction of miR-34 as described in (A). α-tubulin served as a loading control. Results of densitometric analysis are given below the respective bands. (C+D) The indicated CRC cell lines were treated with DOX for 48 hours or left untreated. qPCR analysis of the indicated mRNAs (*CDH1* = *E-cadherin*, *OCLN* = *Occludin*, *FN* = *Fibronectin*). Results represent the mean +/- S.D. (n=3). All significances were calculated with a Student’s t-test. ” * “ : $p < 0.05$. (E) Colo320 cells were transfected either with a control (ctrl) or a miR-34a oligo. qPCR analysis of the mRNA levels of different EMT markers. Results represent the mean with S.D. (n=3). ” * “ : $p < 0.05$.
