# Supplementary material for: Repression of c-Kit by p53 is mediated by miR-34 and is associated with reduced chemoresistance, migration and stemness
Source: Oncotarget. 2013 Aug 6;4(9):1399–415. doi: 10.18632/oncotarget.1202 (PMC3824539; doi:10.18632/oncotarget.1202)
Supplement: Supplementary file 2 [file oncotarget-04-1399-s002.pdf]

Repression of c-Kit by p53 is mediated by miR-34 and is associated with reduced chemoresistance, migration and stemness – Siemens et al

Supplementary Table 1. Primary and secondary antibodies used in this study

Primary Antibodies

Name	clone	order no.	company	Use	Dilution	Source
c-Kit		# A4502	Dako	WB	1:500	rabbit
α-tubulin	DM 1A	# T-9026	Sigma	WB	1:1000	mouse
p53	DO-1	# sc-126	Santa Cruz	WB	1:1000	mouse
p-Erk = Phospho-p44/42 MAPK		# 9101	Cell Signaling	WB	1:500	rabbit
Erk = p44/42 MAPK		# 9102	Cell Signaling	WB	1:500	rabbit

Secondary Antibodies

Name	clone	order no.	company	Use	Dilution	Source
anti-mouse HRP		# W4021	Promega	WB	1:10.000	Goat
anti-rabbit HRP		# A0545	Sigma	WB	1:10.000	Goat

Supplementary Table 2. Plasmids used in this study

Name	ORF/Rep	Reference
pRTR		Jackstadt et al., 2013
pRTR-p53	p53	Siemens et al., 2011
pRTR-miR-34a	miR-34a	Kaller et al., 2011
pRTR-c-Kit	c-Kit	
pGL3-control-MCS		Welch et al., 2007
pGL3-c-Kit	human c-Kit 3´UTR	
pGL3-c-Kit mut1	human c-Kit 3´UTR	
pGL3-c-Kit mut2	human c-Kit 3´UTR	
pGL3-c-Kit mut1 + 2	human c-Kit 3´UTR	
pRL		Pillai et al., 2005

Supplementary Table 3. Oligonucleotides used for cloning and mutagenesis

Designation	Sequence (5' to 3')
human c-Kit 3'UTR fwd	ACCCTGGCATTATGTCCACT
human c-Kit 3'UTR rev	GGGAATATTCAAAAGACATTATTGC
kit 34a site1mut fwd	CCCACAGGAGTGGGAAAACA**G**T**CGG**ATCTTAGTTTGGATTCT
kit 34a site1mut rev	AGAATCCAAACTAAGAT**CCG**A**C**TGTTTTCCCACTCCTGTGGG
kit 34a site2mut fwd	ACTCCCCTTCCTCA**G**T**CGG**CAATATAAAAGGCAAATGTGTAC
kit 34a site2mut rev	GTACACATTTGCCTTTTATATTG**CCG**A**C**TGAGGAAGGGGAGT

Supplementary Table 4. List of qPCR-primers used in this study

Designation	Sequence (5' to 3')
β-actin fwd	TGACATTAAGGAGAAGCTGTGCTAC
β-actin rev	GAGTTGAAGGTAGTTTCGTGGATG
c-Kit fwd	CAGGCAACGTTGACTATCAGT
c-Kit rev	ATTCTCAGACTTGGGATAATC
pri-miRNA-34a fwd	CGTCACCTCTTAGGCTTGGA
pri-miRNA-34a rev	CATTGGTGTCGTTGTGCTCT
Oncostatin M fwd	CACACAGAGGACGCTGCTCA
Oncostatin M rev	ATGCTCGCCATGCTTGGAA
c-Fos fwd	AGAATCCGAAGGGAAGGAA
c-Fos rev	ATCAAGGGAAGCCACAGACA
CD44 fwd	GCCTACTGCAAATCCAAACAC
CD44 rev	GAAGCTCTGAGAATTACTCTGCTG
CD133 fwd	TCCACAGAAATTACCTACATTGG
CD133 rev	CAGCAGAGAGCAGATGACCA
Lgr5 fwd	GCATTTGGAGTGTGTGAGAA
Lgr5 rev	AGGGCTTTCAGGTCTTCCTC
OLFM4 fwd	TGGTCATACAGCTGAAGGAGAGT
OLFM4 rev	GCTTCTCTACCAAGAGAGTCATATTTC
β-catenin fwd	AGCTGACCAGCTCTCTCTTCA
β-catenin rev	CCAATATCAAGTCCAAGATCAGC
Nanog fwd	ATGCCTCACACGGAGACTGT
Nanog rev	AGGGCTGTCCTGAATAAGCA
Nanog P8 fwd	TCCATCCTTGCAAATGTCTTC
Nanog P8 rev	AGGGCTGTCCTGAATAAGCA
BMI-1 fwd	TTCTTTGACCAGAACAGATTGG
BMI-1 rev	GCATCACAGTCATTGCTGCT
Sox2 fwd	TGCGAGCGCTGCACAT
Sox2 rev	TCATGAGCGTCTTGGTTTTCC
CDH1 fwd	CCCGGGACAACGTTTATTAC
CDH1 rev	GCTGGCTCAAGTCAAAGTCC
Vimentin fwd	TACAGGAAGCTGCTGGAAGG
Vimentin rev	ACCAGAGGGAGTGAATCCAG
OCLN fwd	GGCCTCTTGAAAGTCCACCT
OCLN rev	CTGAGAGAGCATTGGTCGAA
FN fwd	CTTTGGTGCAGCACAACTTC
FN rev	TCCTCCTCGAGTCTGAACCA
